# Development and Verification of a Mouse Model for Roux-en-Y Gastric Bypass Surgery with a Small Gastric Pouch

**DOI:** 10.1371/journal.pone.0052922

**Published:** 2013-01-11

**Authors:** Zheng Hao, Zhiyun Zhao, Hans-Rudolf Berthoud, Jianping Ye

**Affiliations:** 1 Antioxidant and Gene Regulation Laboratory, Pennington Biomedical Research Center, Louisiana State University System, Baton Rouge, Louisiana, United States of America; 2 Neurobiology of Nutrition Laboratory, Pennington Biomedical Research Center, Louisiana State University System, Baton Rouge, Louisiana, United States of America; University of Ulster, United Kingdom

## Abstract

Existing mouse models of Roux-en-Y gastric bypass (RYGB) surgery are not comparable to human RYGB in gastric pouch volume for a large or absent gastric volume. The aim of this study was to develop and characterize a mouse RYGB model that closely replicates gastric pouch size of human RYGB surgery of about 5% of total gastric volume. We established this model in diet-induced obese (DIO) mice of C57BL/6J. This surgery resulted in a sustained 30% weight loss, entirely accounted for by decreased fat mass but not lean mass, compared to sham-operated mice on the high fat diet. Compared to sham-operated mice, energy expenditure corrected for total body weight was significantly increased by about 25%, and substrate utilization was shifted toward higher carbohydrate utilization at 8 weeks after RYGB when body weight had stabilized at the lower level. The energy expenditure persisted and carbohydrate utilization was even more pronounced when the mice were fed chow diet. Although significantly increased during daytime, overall locomotor activity was not significantly different. In response to cold exposure, RYGB mice exhibited an improved capacity to maintain the body temperature. In insulin tolerance test, exogenous insulin-induced suppression of plasma glucose levels was significantly greater in RYGB mice at 4 weeks after surgery. Paradoxically, food intake measured at 5 weeks after surgery was significantly increased, possibly in compensation for increased fecal energy loss and energy expenditure. In conclusion, this new model is a viable alternative to existing murine RYGB models and the model matches human RYGB surgery in anatomy. This model will be useful for studying molecular mechanisms involved in the beneficial effects of RYGB on body weight and glucose homeostasis.

## Introduction

Among bariatric surgeries, Roux-en-Y gastric bypass (RYGB) is most effective in the treatment of obesity, type-2 diabetes, and other comorbidities of the obese state [1], [2], [3]. Although a number of hypotheses have been proposed [4], [5], the exact mechanism(s) for these beneficial effects of RYGB surgery remain unknown. Rodent models are increasingly used, as they allow more invasive techniques necessary to carry out mechanistic studies [6], [7], [8]. The mouse will be particularly important, as transgenic models allow direct testing of specific hormone or signaling molecule [9], [10]. However, the existing mouse models vary greatly in the surgical detail and do not closely mimic typical RYGB surgery in humans. Specifically, the first mouse RYGB model was made in diet-induced obese (DIO) mice, in which the entire stomach was left intact while the pyloric sphincter was ligated, and the jejunum was anastomosed to the greater curvature of the stomach [7], [9]. A second mouse RYGB model in DIO mice involved anastomosing the jejunum directly to the esophagus after ligating the cardia, leaving no gastric pouch at all [11]. It was mentioned in the study that forming a gastric pouch resulted in excessive mortality, likely due to inadequate gastric smooth muscle contraction in the forestomach causing obstruction [11]. A third mouse model published most recently uses a metal clip to form a relatively large (∼30%) gastric pouch that includes the entire murine forestomach, to which the jejunum is then anastomosed [10], [12]. Thus, none of the existing mouse models closely replicates RYGB surgery in humans, which generally has a pouch size of about 5% of the gastric volume.

The aim of our study was, therefore, to develop and verify a mouse model of RYGB that is based on a pouch size of about 5% of the total stomach volume, similar to typical RYGB surgery in humans, which could then be used in the mechanistic studies using genetically altered mice. By taking particular care to preserve the vasculature near the cardia of the stomach, we were attempting to reduce acute surgical trauma to facilitate intake of solid food soon after surgery. Male C57/BL6J mice were made obese by feeding them a high-fat diet for 12 weeks and then used in the surgery. To verify the effectiveness of the surgery, we monitored body weight for 8 weeks after surgery and measured body composition, food intake, energy expenditure, body temperature response to cold exposure, locomotor activity, and insulin tolerance at specific time points after surgery.

## Methods

### Diet-induced obese (DIO) mice

All experiments were performed in compliance with and were approved by the Institutional Animal Care and Use Committee (IACUC) of Pennington Biomedical Research Center. Animals were housed under conventional conditions at the Pennington Animal Facility. C57BL/6J mice from Jackson Laboratory were fed a high fat diet (HFD, D12331 diet, 58% calories from fat, Research Diets Inc.) at 8 weeks-old for 12 weeks. At the time of surgery, they weighed 46±5 g. All animals were fed ad libitum with HFD before and after surgery, except in the energy expenditure experiment where mice were fed the high fat diet for the first 3 days and then chow diet for the subsequent 3 days in the metabolic chamber. In addition, mice were given access to chow and high-fat diet for about 2–3 days prior to surgery.

### Surgical Procedures

Animals were fasted 4 to 6 hours before operation. Anesthesia was induced and maintained with isoflurane. Standard aseptic procedures were used throughout. For RYGB, a midline incision starting from the xiphoid process was made and the perigastric ligaments were ligated and cut to release the stomach.

**Figure 1:**
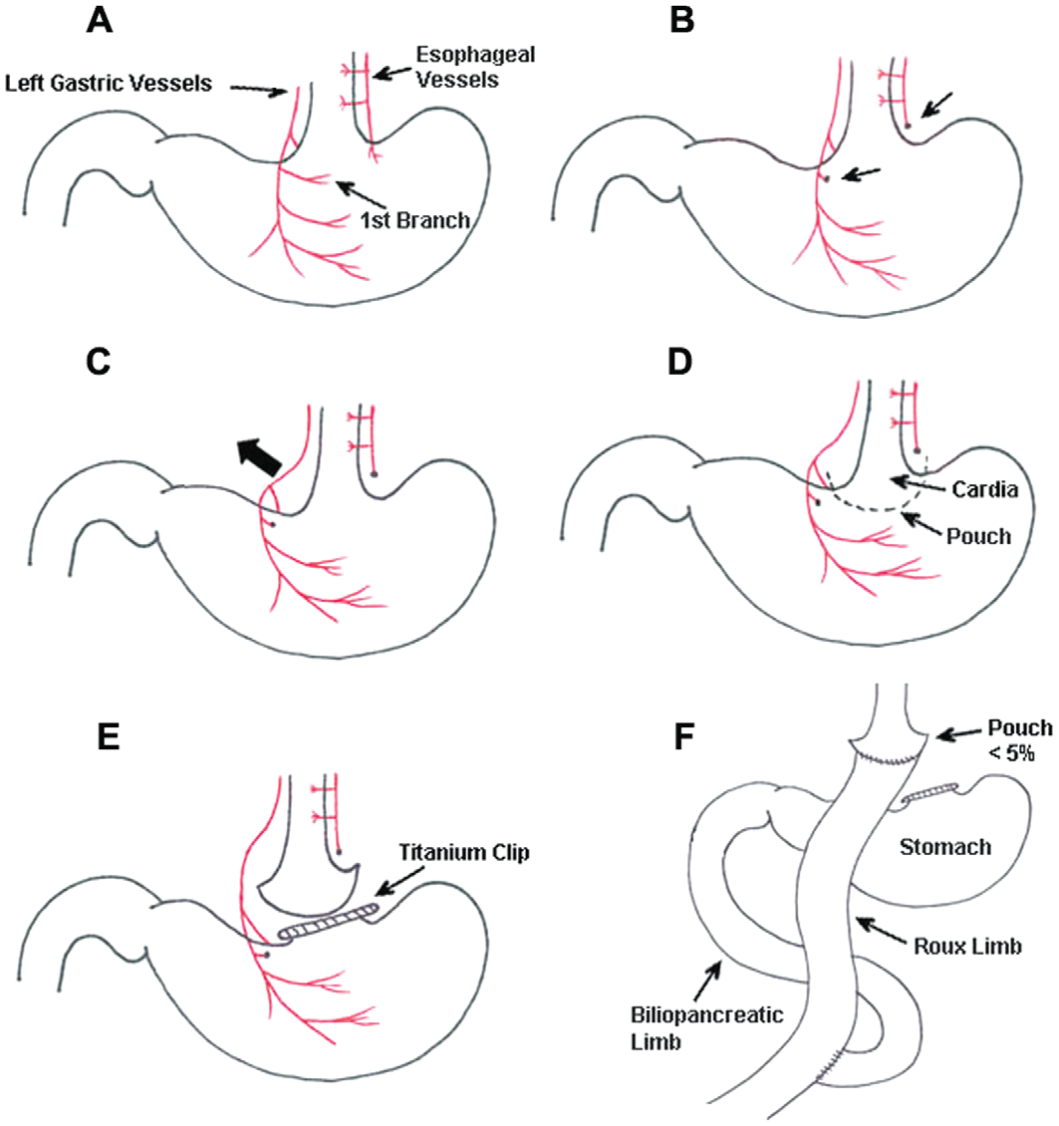
Schematic illustration of generating the small gastric pouch (<5% of total gastric volume). (A) Perigastric ligaments are ligated and cut to release the stomach. The arrow indicates the left gastric vessels and esophageal vessels. (B) The first branch of the left gastric vessels and the esophageal vessels are ligated and cut. (C) The left gastric vessels are bluntly separated from the cardia to make room for pouch operation without impairing the gastric blood supply. (D) and (E) A titanium clip is applied to the stomach with care not to impinge on the left gastric vessel bundles, and the stomach is transected right above the clip. (F) The small gastric pouch is anastomosed to the cut jejunum.

**Figure 2:**
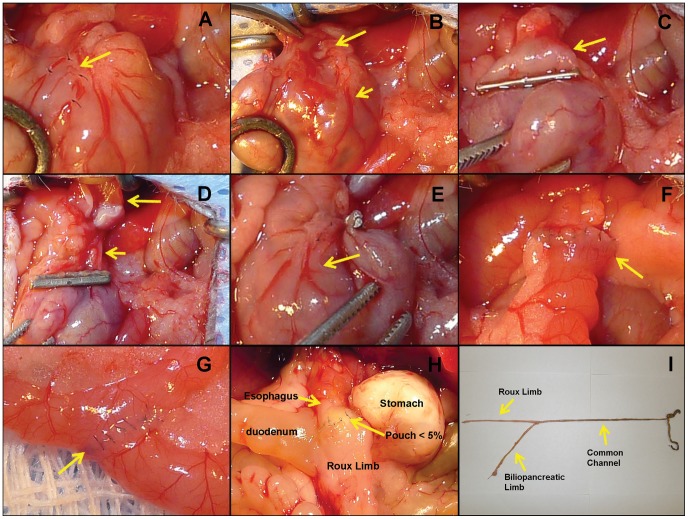
Series of images depicting small pouch Roux-en-Y gastric bypass surgery in the mouse. (A) The first 2 branches of the posterior left gastric vessels were ligated and cut. (B) The left gastric vessel bundles were separated from esophagus. The short arrow indicates the posterior left gastric vessels. The long arrow shows the gap between left gastric vessels and the cardia. (C) A titanium clip was applied to make the small gastric pouch (arrow). (D) The stomach was transected immediately above the clip, leaving the left gastric vessels intact (short arrow). The long arrow indicates the small gastric pouch. (E) The intact left gastric vessels (arrow point) guarantees normal blood supply of the stomach. (F) The arrow points to the completed gastrojejunostomy. (G) The arrow points to the completed jejunojejunostomy. (H) State of the gastric pouch at 3 weeks after surgery with the esophagus at the top (short arrow) and the small gastric pouch below (large arrow), clearly demonstrating that there was no expansion of the pouch after eating solid food for 3 weeks. (I) Picture showing the relative lengths of the Roux, biliopancreatic, and common limbs after RYGB surgery.

As illustrated schematically in Fig. 1 and photographically in Fig. 2, there are major steps in the surgical procedure. The first step is to prepare the cardia region of the stomach for placement of a clip and transection of the stomach with minimal bleeding. To this end, the first one or two branches of both the anterior and posterior left gastric vessels, as well as the esophageal vessels, were ligated and cut (Fig. 1, A and B; Fig. 2A), and the main anterior and posterior left gastric vessel bundles were separated from the serosa by gently grasping them with serrated forceps and blunt dissection (Fig. 1, C and D). This step is crucial, as it guarantees normal blood supply to the separated stomach.

The second step is to make a small gastric pouch with a volume of about 5% of the total gastric volume, the standard pouch size in human RYGB. To this end, a titanium clip (medium size titanium hemostatic clip; Vitalitec Inc., Plymouth, MA 023060) was applied across the area just below the cardia, from which the left gastric vessel bundles had been lifted or removed (Fig. 1, D and E, Fig. 2D), and the pouch was separated from the rest of the stomach by cutting immediately above the clip with a pair of straight microscissors. Importantly, because the blood supply was previously removed or re-routed, this resulted in negligible bleeding. The pouch was then anastomosed with the open end of the jejunum by means of 16–18 interrupted stiches with 11-0 nylon suture (Fig. 1F, and Fig. 2F). In pilot experiments, the pouch was examined 3 weeks after surgery, no expansion was observed in the pouch size (Fig. 2H). Finally, the stomach was re-inserted into the upper left abdominal cavity.

The third step comprises the jejuno-jejunostomy that determines the lengths of the Roux and common limbs. In pilot experiments, we determined the length of the small intestine in vivo in chow-fed and high fat diet-induced obese mice, by using the 1 cm long tip of a pair of angled forceps. We found that the average length of the small intestine from the ligament of Treitz to the cecum is about 22 cm in chow-fed mice and about 20 cm in long-term DIO C57/BL6J mice. From this, we determined that in obese mice, the optimal lengths are about 5–6 cm for the Roux limb and 5–6 cm for the biliopancreatic limb. Therefore, the jejunum was transected about 2 cm distal to the ligament of Treitz, and the cut ends were sterilized with 5% povidone iodine. The distal end was brought up to the stomach pouch for end-to-end anastomosis as described above. For the jejuno-jejunostomy, a longitudinal slit was made on the antimesenteric side of the jejunum at 6 cm distal to the site of gastrojejunostomy, and the proximal end of the jejunum was joined in an end-to-side anastomosis using 11-0 nylon suture in an interrupted fashion. This resulted in a common limb consisting of the distal jejunum and the ileum of about 12 cm (Fig. 2I). Before closing of the abdominal cavity, the intestine was arranged in an “S” position to avoid intestinal obstruction. In the abdominal wall, the muscular layer was closed using interrupted 11-0 nylon suture, and the skin was closed using interrupted 5-0 nylon suture. All sutures were purchased from the Chenghe Microsurgical Equipment Company (Ningbuo City, Zhejiang Province, China).

For the sham operation, the perigastric ligaments were cut, and then a 3 mm incision was made in the stomach wall and closed with a titanium clip. In addition, the jejunum was transected 2 cm distal to the ligament of Treitz, and the two cut ends were anastomosed using 11-0 nylon suture in an interrupted fashion. These procedures are designed to inflict at least some of the tissue damage and similar surgical trauma as by the real RYGB, but not to change the nutrient flow through the GI-tract, which is hypothesized to cause its beneficial effects.

### Postoperative care

For the first 24 hours after the operation, the mice were put in regular shoe box cages without bedding on a heating pad set at 35°C. The mice were given 0.7 ml of 5% dextrose subcutaneously and carprofen (5 mg/kg, s.sc) for analgesia, immediately after surgery. Water and normal chow was made available after recovery from anesthesia. On the day after surgery, mice were moved to shoe box cages with regular bedding, and high-fat diet was provided 3 days after surgery.

### Measurement of body weight, body composition, and food intake

Body weight was measured daily, and body composition was measured at the end of the observation period at 8 weeks by NMR. Food intake was measured at 5 weeks after surgery. Pre-weighed high-fat diet was given directly into the shoe box cages and the remaining diet was measured 3 days later. Average daily intake was calculated with taking spillage into account.

### Measurement of energy expenditure and locomotor activity

Ten DIO mice were used in this study after surgery. Body composition (NMR), energy expenditure, and cold response were tested between 6–9 weeks after surgery. Those metabolic tests were conducted using protocols described elsewhere [13]. In the 6 day energy expenditure assay, mice were on HFD for the first 3 days and then chow diet for the next 3 days in the metabolic chamber. Data were collected and used in this study after adaptation to the chambers for 1 day.

### Measurement of insulin sensitivity

Insulin tolerance was performed at 4 weeks after surgery to determine insulin sensitivity. The test was conducted with peritoneal injection of insulin at 0.7 U/Kg after 4 h fasting.

### Statistical analysis

In this study, values are presented as mean ± SEM. Student's t test was used in most data analysis for body weight, insulin tolerance, energy expenditure, substrate utilization, and body temperature. Two-way ANOVA was used in data analysis for diet and surgery effects on energy expenditure. P values<0.05 were considered significant. All statistical tests were two-tailed.

## Results

After establishing the basic surgical approach in carcasses and non-survival surgeries, RYGB was carried out in an additional 34 mice to determine optimal postsurgical dietary requirements and verify the intended pouch size. Fifteen (44%) of these mice died or had to be euthanized within the first week after surgery, mainly due to complications caused by mechanical obstruction at the end-to-side jejuno-jejunostomy. To reduce this risk, we used 11-0 nylon suture with 16–20 interrupted stitches and employed as little bowel wall as possible (<0.5 mm on each side) in the anastomosis (Fig. 2G).

RYGB was then performed in a total of 5 mice, and sham surgery in 6 mice in this study. One of the five RYGB mice exhibited lack of appetite and rapid weight loss and was euthanized 5 days after surgery. Part of the Roux limb had formed adhesions to the gastrojejunostomy leading to mechanical obstruction. The remaining 4 RYGB mice recovered without problems and were followed for 8 weeks after surgery, yielding the results reported here. The operating time for each mouse was 80–90 minutes, and the mice were able to eat some solid diet within 24 hours after surgery without complications. All 6 sham-operated mice recovered quickly.

### Small pouch RYGB results in sustained reduction in body weight and fat mass without affecting lean mass

**Figure 3:**
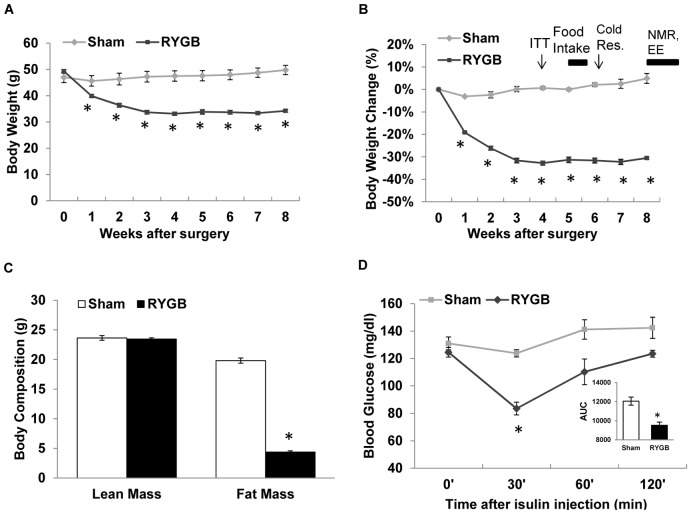
Small pouch RYGB reduces fat mass and improves insulin tolerance in DIO mice. (A) Body weight in gram. (B) Body weight change in percentage after RYGB or sham surgery. Note timing of insulin tolerance test (ITT) and cold response test, as well as food intake, body composition (NMR), and energy expenditure (EE) measurements indicated at the top. (C) Fat mass and lean mass at 8 weeks after RYGB or sham surgery. Note the significant reduction in fat mass but not lean mass after RYGB. (D) Insulin tolerance test showing significantly greater decrease of plasma glucose levels after administration of insulin (0.7 Unit/Kg, i.p.) in RYGB mice compared to sham-operated mice, at 8 weeks after surgery. Area under the curve (AUC) for 0–120 min is shown in the insert bar figure. Means ± SEM (RYGB n = 4, Sham n = 6). * p<0.05.

RYGB resulted in rapid loss of body weight over the first 3 weeks (Fig. 3, A and B). RYGB mice lost about 20% of body weight during the first 2 weeks and about 30% after 3 weeks. After the third week, body weight remained stable for another 5 weeks. Body composition analysis carried out at 8 weeks after surgery revealed that body weight loss was entirely due to loss of fat mass, with no difference in lean mass between RYGB and sham-operated mice (Fig. 3C). In sham-operated mice, body weight was only slightly reduced during the first two weeks. Then the weight recovered at week 3 and exceeded the basal level thereafter (Fig. 3, A and B). The sham group gained 5% body weight above the basal level at the end of 8 weeks. The weight loss after RYGB was entirely due to loss of fat mass (∼15 g) but not lean mass (Fig. 3C), as measured by NMR at 8 weeks post-surgery.

### Small pouch RYGB improves insulin action

Insulin sensitivity was examined in the mice at 4 weeks after surgery with the insulin tolerance test. The result suggests that RYGB mice exhibit significant improvement in insulin sensitivity (Fig. 3D). The significant difference was observed in glucose levels and in the area under the cure in the insert bar figure.

### After the rapid weight loss phase, RYGB mice show increased energy expenditure and food intake

**Figure 4:**
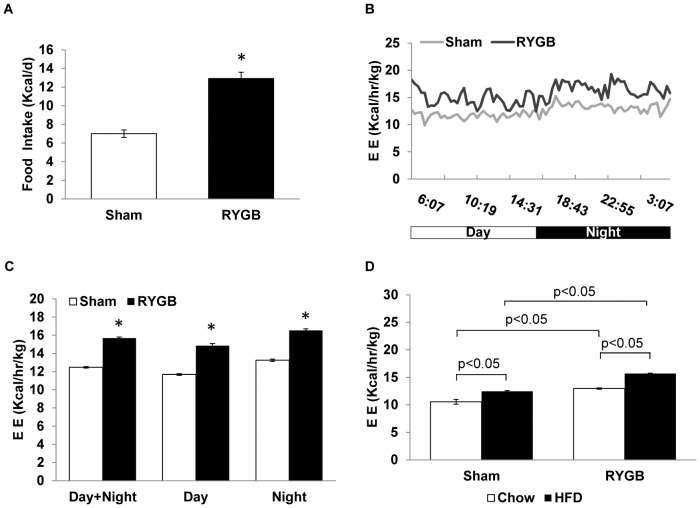
Small pouch RYGB increases food intake and energy expenditure (EE) in DIO mice. (A) Average daily high-fat diet intake measured over a period of 3 days at 5 weeks after RYGB or sham surgery. (B) Energy expenditure at 8 weeks after surgery as measured by indirect calorimetry normalized to total body mass. B. Pattern of energy expenditure over 24 h period. (C) Energy expenditure of RYGB and sham-operated mice measured over two consecutive days and shown as average rate per hour for the entire diurnal cycle and separately for day and night on the high fat diet. (D) Comparison of energy expenditure on high-fat diet (HFD, black bars) and on chow (white bars) in RYGB and sham-operated mice. Means ± SEM (RYGB n = 4, Sham n = 6). * p<0.05, based on the student *t* test or two-way ANOVA test.

Somewhat unexpectedly, we found that RYGB mice increased intake of high-fat diet by almost 80% compared to sham-operated mice, measured 5 weeks after surgery (Sham: 7.01±0.40; RYGB: 12.93±1.33 Kcal/day, p<0.01) (Fig. 4A). Increased energy intake during this weight-stable phase suggests that the mice were compensating for energy losses in the feces and increased energy expenditure. Although we did not measure fecal energy loss, the soft and smelly feces strongly suggest malabsorption and substantial loss of energy, probably mainly from fat, in the feces.

In addition, we found that energy expenditure normalized to total body mass was significantly enhanced by about 26% in RYGB mice compared to sham-operated mice (Sham: 12.47±0.12; RYGB: 15.69±0.08 Kcal/kg/day, p<0.01) (Fig. 4, B and C). Energy expenditure was significantly increased during both the light (6:00am–6:00pm) and dark (6:00 pm–6:00 am) phase (Fig. 4C) and was also observed during chow-feeding (Fig. 4D). When energy expenditure was corrected for lean mass, it was no longer different between RYGB and sham surgery.

**Figure 5:**
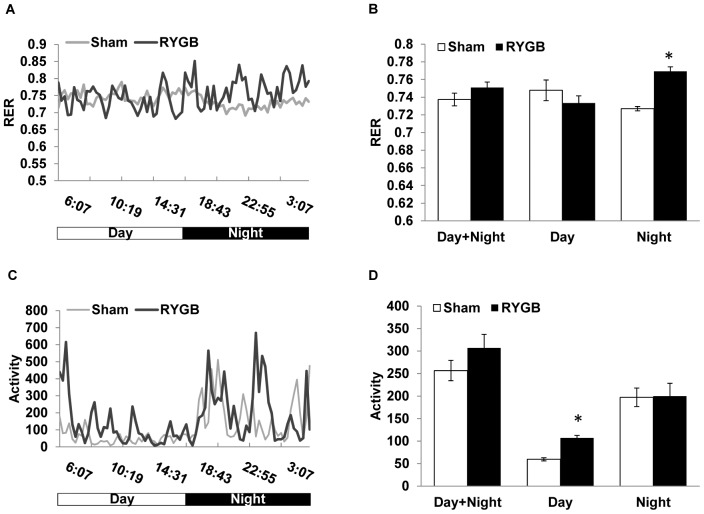
Effects of small pouch RYGB on respiratory exchange ratio (RER) and locomotor activity. These tests were conducted at 9 weeks after surgery in mice fed a high-fat diet. (A) Diurnal pattern of RER. (B) Average RER for the entire diurnal cycle and separately for day and night. Note the significantly higher RER of RYGB mice during the dark period. (C) Diurnal pattern locomotor activity. (D) Average locomotor activity for the entire diurnal cycle and separately for day and night. Note the significantly higher activity of RYGB mice during the light period. Means ± SEM (RYGB n = 4, Sham n = 6). * p<0.05.

The respiratory exchange ratio (RER) was measured to obtain information regarding substrate utilization for mitochondrial ATP production, with high RER values indicating preferential carbohydrate utilization and low RER values indicating preferential fat utilization. At 8 weeks after surgery, when all excess fat mass had been lost and body weight was stable, RER during the dark phase was significantly higher in RYGB compared to sham-operated mice (Fig. 5, A and B), suggesting that RYGB mice depend slightly more on carbohydrates for ATP production compared to sham-operated mice. Because light-phase RER was slightly lower, there was no significant difference in 24 h RER between RYGB and sham-operated mice.

The physical activity level (locomotor activity) was examined to determine its possible role in elevated energy expenditure. Locomotor activity was significantly higher in RYGB compared to sham-operated mice during the light phase (Fig. 5, C and D), probably reflecting changes in the pattern of food intake, with a shift to a higher proportion of intake during the light phase (day time) to compensate for reduced meal size after RYGB. However, because daytime activity accounts for only a small portion, total daily activity was not significantly different.

**Figure 6:**
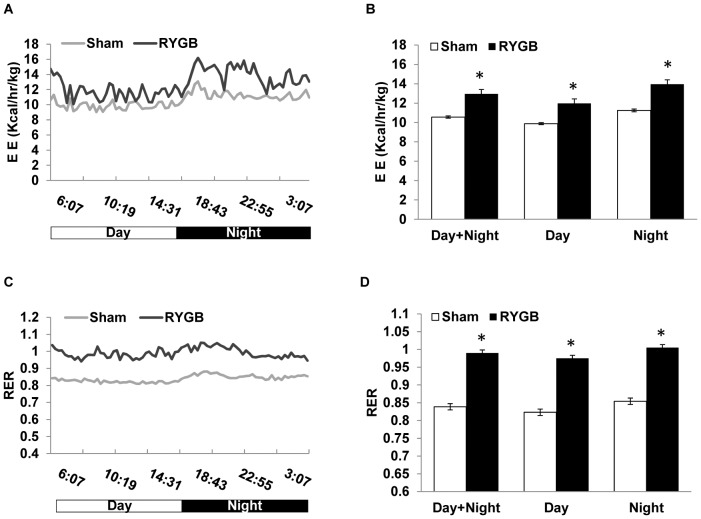
Small pouch RYGB increases energy expenditure and respiratory exchange rate (RER). These tests were conducted in DIO mice on chow diet. (A) Diurnal pattern of energy expenditure. (B) Average energy expenditure for the entire diurnal cycle and separately for day and night. The test was conducted in DIO mice at 9 weeks after RYGB or sham surgery and fed normal chow diet during testing. Note the significantly higher energy expenditure during both light and dark periods. (C) Diurnal pattern of RER. (D) Average RER for the entire diurnal cycle and separately for day and night. Note the significantly higher RER of RYGB mice for both day and night. Means ± SEM (RYGB n = 4, Sham n = 6). * p<0.05.

To measure energy expenditure and physical activity in mice on low-fat diet, we switched animals in the Oxymax system after 3 days of high-fat to 3 days of regular chow diet and analyzed the data for the last two days after the mice had adapted to the new diet. Similar to the period of high-fat feeding, energy expenditure corrected for total body mass was significantly higher by about 23% in RYGB compared to sham-operated mice under conditions of low-fat feeding, and the effect was present during both the light and dark periods (Fig. 6, A and B).

**Figure 7:**
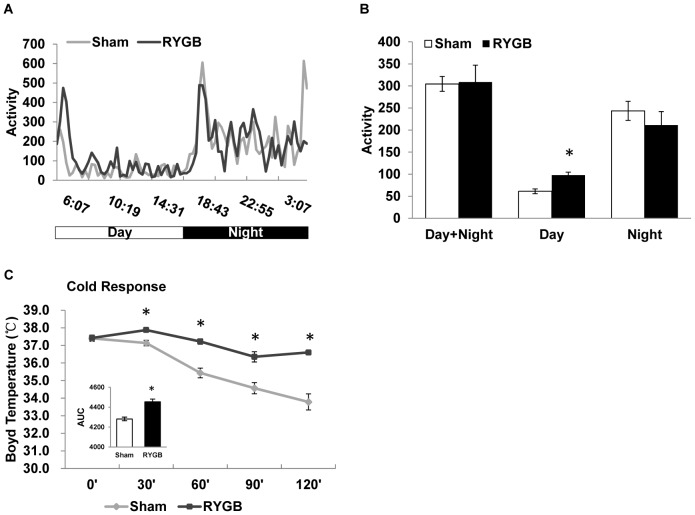
Effects of RYGB on locomotor activity and response to cold exposure in mice fed chow diet. (A) Diurnal pattern of locomotor activity. (B) Average locomotor activity for the entire diurnal cycle and separately for day and night. Note the significantly higher activity of RYGB mouse during the light period. (C) Cold exposure-induced change in body temperature of DIO mice at 6 weeks after RYGB or sham surgery and fed normal chow diet during testing. Area under the curve (AUC) is shown in the inset bar figure. Note that RYGB mice are less able to resist cold exposure compared with sham-operated mice. Means ± SEM (RYGB n = 4, Sham n = 6). * p<0.05.

However, unlike under high-fat feeding, the increase in RER was much more robust in RYGB mice under low-fat (high-carbohydrate) feeding and extended throughout day and night (Fig. 6, C and D), suggesting that RYGB mice depend more on carbohydrate for ATP production compared to sham-operated mice. Finally, we found that under low-fat feeding conditions, locomotor activity was only significantly increased during the light phase (day time), but not over the dark phase (night) or entire day (Fig. 7, A and B).

### Small pouch RYGB mice exhibit increased cold tolerance

RYGB mice had a higher energy metabolism on both HFD and chow diet. It is not known if brown fat contributes to energy metabolism. Brown fat produces heat from glucose and fatty acids in the maintenance of body temperature. To address this question, we conducted a cold response experiment to test the brown fat function at 6 weeks after surgery. In the cold room of 4°C, the body temperature dropped significantly over 2 hours in the sham group (Fig. 7C). The drop was significantly less in RYGB mice (Fig. 7C). The difference in temperature was observed at every time point during the experiment. The data suggests that brown fat in RYGB mice has a stronger activity in heat production, which is consistent with the elevated energy expenditure.

## Discussion

Existing mouse models for RYGB surgery have either no gastric pouch at all [11], a relatively large gastric pouch (30%–50% of total gastric volume) [10], [12], or no reduction of stomach volume [7], [9]. One likely reason that smaller gastric pouches in mouse RYGB models have not been reported is the technical difficulty of the surgery. We overcame this problem by carefully lifting the left gastric artery, allowing transection of the stomach near the cardia, and including about 5% of the total gastric volume in the pouch. Our RYGB model thus replicates human RYGB surgery with respect to gastric pouch size and lengths of Roux and biliopancreatic limbs. On a provisionary note, one chow-fed female C57/BL6J mouse with RYGB surgery gave birth to a litter of pups at 4 months after surgery, further demonstrating the excellent health of mice undergoing our surgical protocol.

### Surgical Implications and recommendations

In our pilot studies, we found that after RYGB mice often died or had to be euthanized because of intestinal obstruction at the jejuno-jejunostomy, resulting in a mortality rate of more than 50%. Using 11-0 nylon sutures in an interrupted fashion, significantly reduced such obstruction and mortality. Failure to properly arrange the Roux limb in an “S” fashion appeared to increase the risk for the intestinal obstruction. Obstruction usually manifests itself on the first day after surgery with secretion from the eyes and lethargy. If there are no postsurgical complications, the mice look healthy and alert, and are able to eat solid diet within 24 hours. In most other mouse and rat models, liquid diet has to be used for up to 10 days to prevent obstruction, and animals have to be carefully adapted to solid foods [12], [14]. One possible explanation is that the smooth muscle of the stomach pouch is too weak to push solid food into the Roux limb, particularly when the foramen of the gastro-jejunostomy is kept small [11]. In this case, the pouch over-distends and then food is eventually pushed back into the esophagus. We believe that this is not a major problem in our mouse model because the stomach pouch is kept small and because of increased elasticity of the anastomosis due to using interrupted 11-0 nylon sutures and including as little gastric and jejunal wall as possible in the anastomosis.

Another postsurgical complication often observed in mice and rats is foamy saliva smeared over the snout. The saliva often contains traces of blood, and the affected animals exhibit excessive mouth wiping. We believe that this is mainly due to excessive stretch of the esophagus when preparing the gastric pouch and gastro-jejunal anastomosis.

Additional recommendations according to our experience are as follows: 1) Providing regular chow for 2–3 days before surgery appears to reduce the risk of complications. This beneficial effect may be due to the higher fiber content of chow, which is known to stimulate gastrointestinal motility [15], which may be quite low in the animals on high-fat diet for several weeks. 2) Carprofen is better than Buprenorphine for analgesia, as it produces less inhibition of gastrointestinal motility (refs). 3) Bedding or any other chewable material should be avoided in the cage for the first 2 days after surgery. We found that mice tend to chew, or even eat, the bedding materials after surgery.

### Mechanistic implications

We show here that the model replicates some, but not all, physiological changes reported after RYGB in humans [16], [17], [18] and rodents [8], [12], [19], [20]. The 30% sustained weight loss, almost complete loss of excess fat mass, increased energy expenditure, and improved glucose homeostasis are similar to reports in other preclinical and clinical studies, but the quite significantly increased food intake and the complete sparing of lean mass have not been reported in human studies or in most rodent studies. Only one other mouse study reported significantly increased food intake, but of a much lower magnitude [12]. In that study, an in-depth energy balance analysis over the first 6 weeks after surgery clearly demonstrated that large pouch RYGB mice lost a considerable amount of energy in the feces and in higher resting energy expenditure, suggesting that the relative hyperphagia of about 5% was an attempt to overcome these energy losses. Because energy intake was only reported cumulatively for 6 weeks, the true hyperphagia during the weight-stable phase is likely underestimated in that study [12] and may have been closer to our findings of about 84% higher intake in RYGB compared to sham-operated mice.

The mouse model also differs from human RYGB, in that the weight loss is much more rapid in our and other mouse models, with about 20–30% weight lost within two weeks after surgery, whereas weight loss in humans is much more protracted over about 12 months. This rapid weight loss makes it difficult to study the weight-loss-independent mechanisms for improvement of glucose homeostasis. We found evidence for improved insulin-sensitivity by measuring the ability of insulin to lower plasma glucose at 4 weeks after surgery, when maximal weight loss had already occurred. We are not able to ascribe it to weight-independent mechanisms for insulin sensitization in rodent models, and pair-feeding/weight-matching strategies will be necessary in future experiments.

In conclusion, we describe and verify a novel model of RYGB in diet-induced obese mice with the size of the gastric pouch closely matching human surgery. The model is characterized by increased energy expenditure in the face of increased food intake and resulting in sustained loss of excess fat and improvement in insulin sensitivity. The model will be valuable to test candidate mechanisms in genetically altered animals.
